# Long noncoding RNA NRON contributes to HIV-1 latency by specifically inducing tat protein degradation

**DOI:** 10.1038/ncomms11730

**Published:** 2016-06-13

**Authors:** Jun Li, Cancan Chen, Xiancai Ma, Guannan Geng, Bingfeng Liu, Yijun Zhang, Shaoyang Zhang, Fudi Zhong, Chao Liu, Yue Yin, Weiping Cai, Hui Zhang

**Affiliations:** 1 Institute of Human Virology, Zhongshan School of Medicine, Sun Yat-sen University, Guangzhou, Guangdong 510080, China; 2 Key Laboratory of Tropical Disease Control of Ministry of Education, Zhongshan School of Medicine, Sun Yat-sen University, Guangzhou, Guangdong 510080, China; 3 Department of Infectious Diseases, Guangzhou 8th People's Hospital, Guangzhou, Guangdong 510060, China

## Abstract

Long noncoding RNAs (lncRNAs) play multiple key regulatory roles in various cellular pathways. However, their functions in HIV-1 latent infection remain largely unknown. Here we show that a lncRNA named NRON, which is highly expressed in resting CD4^+^ T lymphocytes, could be involved in HIV-1 latency by specifically inducing Tat protein degradation. Our results suggest that NRON lncRNA potently suppresses the viral transcription by decreasing the cellular abundance of viral transactivator protein Tat. NRON directly links Tat to the ubiquitin/proteasome components including CUL4B and PSMD11, thus facilitating Tat degradation. Depletion of NRON, especially in combination with a histone deacetylase (HDAC) inhibitor, significantly reactivates the viral production from the HIV-1-latently infected primary CD4^+^ T lymphocytes. Our data indicate that lncRNAs play a role in HIV-1 latency and their manipulation could be a novel approach for developing latency-reversing agents.

## Abstract (web summary)

Long noncoding RNAs have a wide range of physiological functions, though their role in viral infection and latency is poorly understood. Here the authors show a lncRNA NRON can induce degradation of HIV-1 protein Tat, potentially contributing to latent infection.

## Graphical abstract

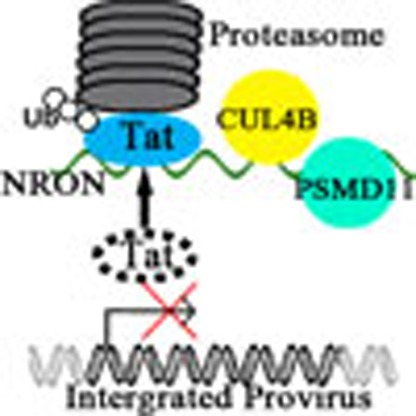

In recent years, tens of thousands of human long noncoding RNAs (lncRNAs) have been annotated by genome-wide signature analysis or multiple deep-sequencing technology^1^,^2^,^3^. With increased reports suggesting their functionality, it has been realized that lncRNAs are more than transactional ‘noise'^4^. LncRNAs can bind to various proteins, DNAs or RNAs to form functional complexes^5^,^6^, and participate in multiple cellular processes including epigenetic modification^7^,^8^,^9^,^10^, transcriptional^11^,^12^ and post-transcriptional regulation^13^,^14^,^15^,^16^ of gene expression, signal transduction^17^, transportation^18^ and many others^19^. LncRNAs have been implicated in differential physiological and pathological processes, such as stemness maintenance^20^, organ development^13^, cancer progression^9^, immune regulation^21^ and pathogen infection^22^,^23^.

HIV-1 latent infection in resting CD4^+^ T lymphocytes is the major obstacle to eradicate the virus in patients after suppressive combination antiretroviral therapy (cART)^24^,^25^,^26^. Because of its presence, the life-long cART is required, otherwise the viremia will rapidly rebound. HIV-1 latency could be due to deficiency of transcriptional factors such as nuclear factor-kappa B (NF-kB) or nuclear factor of activated T-cells (NFAT)^27^,^28^, lack of the viral accessory protein Tat^29^ or condensed chromatin structure and epigenetic regulation^30^,^31^,^32^,^33^. In addition, a cluster of microRNAs including miR-28, miR-125b, miR-150, miR-223 and miR-382, which are enriched in resting CD4^+^ T lymphocytes, target the 3′-untranslated repeat of HIV-1 mRNA to restrict viral gene expression, and subsequently contribute to HIV-1 latency^34^.

Here we investigated the role of lncRNAs in HIV-1 latent infection, and have identified a lncRNA NRON, which potentially contributes to HIV-1 latent infection. NRON RNA significantly inhibits the HIV-1 promoter activity and decreased the cellular abundance of viral transactivator protein Tat. Further mechanism studies suggest that Tat protein is linked to the ubiquitin/proteasome components CUL4B and PSMD11 by NRON, and then degraded. Depletion of NRON RNA, especially in combination with a latency-reverse agent SAHA, robustly reactivated the latently infected proviruses both in an *in vitro* latency model and primary resting CD4^+^ T lymphocytes isolated from infected individuals receiving suppressive cART. Our data indicated that NRON plays a role in HIV-1 latency maintenance, and it could be a new target for reversing viral latency.

## Results

### LncRNA NRON represses HIV-1 replication

**Figure 1:**
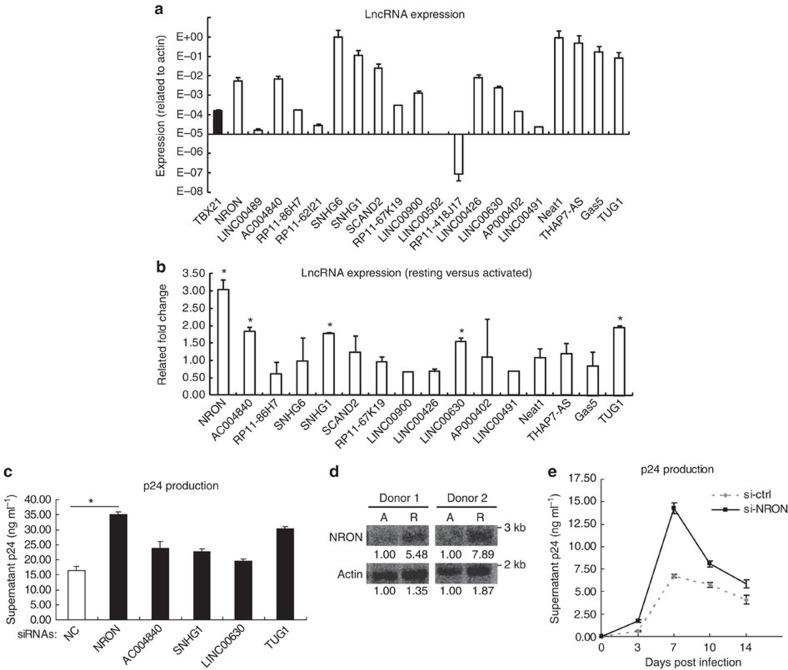
LncRNA NRON represses HIV-1 replication. (**a**) The expression levels of lncRNAs in activated primary CD4^+^ T lymphocytes were detected with real-time qRT–PCR. The expression of *T-bet* was detected as positive control (*n*=3). (**b**) Real-time qRT–PCR detection of the lncRNAs expression level differences between the resting and activated primary CD4^+^ T lymphocytes from a same donor (*n*=3). (**c**) The activated primary CD4^+^ T lymphocytes were transfected with siRNAs against indicated lncRNAs or nonspecific control and were infected with HIV-1_NL4-3_ viruses. HIV-1 productions in the cultures were detected by p24 ELISA at 7 days post infection (*n*=3). (**d**) Northern blotting detection of NRON expression in the resting (R) or activated (A) primary CD4^+^ T lymphocytes from the same donors. Numbers indicated the fold change related to control. (**e**) The activated primary CD4^+^ T lymphocytes were transfected with siRNAs against NRON or nonspecific control and were infected with HIV-1_NL4-3_ viruses. HIV-1 productions in the cultures were detected with p24 ELISA at indicated time points post infection (*n*=3). The results in **a**–**c**,**e** show mean±s.d. (error bars). *P<0.05, Student's unpaired *t-*test.

To determine whether any lncRNA was involved in HIV-1 replication and latency, we selected several lncRNAs that were previously reported to be highly expressed in human immune system including the thymus^18^, lymph nodes and white blood cells^35^, and examined their expression in human primary CD4^+^ T lymphocytes with real-time quantitative reverse transcriptase–PCR (qRT–PCR). Some lncRNAs were highly expressed in comparison with the mRNA level of the control gene *T-bet*, which was reported to be expressed in CD4^+^ T lymphocytes^36^ (Fig. 1a). A few of the abundant lncRNAs showed significantly higher expression levels in resting CD4^+^ T lymphocytes than those in activated cells (Fig. 1b). On screening with a group of lncRNA-specific short interfering RNA (siRNA) pools of two or three siRNAs for one target lncRNA to reduce off-target effects, we found that several lncRNAs including NRON and TUG1 affected the replication of HIV-1 (Fig. 1c and Supplementary Fig. 1). The depletion of NRON, which exerted a definitely higher expression level in resting CD4^+^ T lymphocytes through Northern blotting detection (Fig. 1d), significantly enhanced the viral replication in a time-course study (Fig. 1e).

### NRON specifically represses HIV-1 transcription

**Figure 2:**
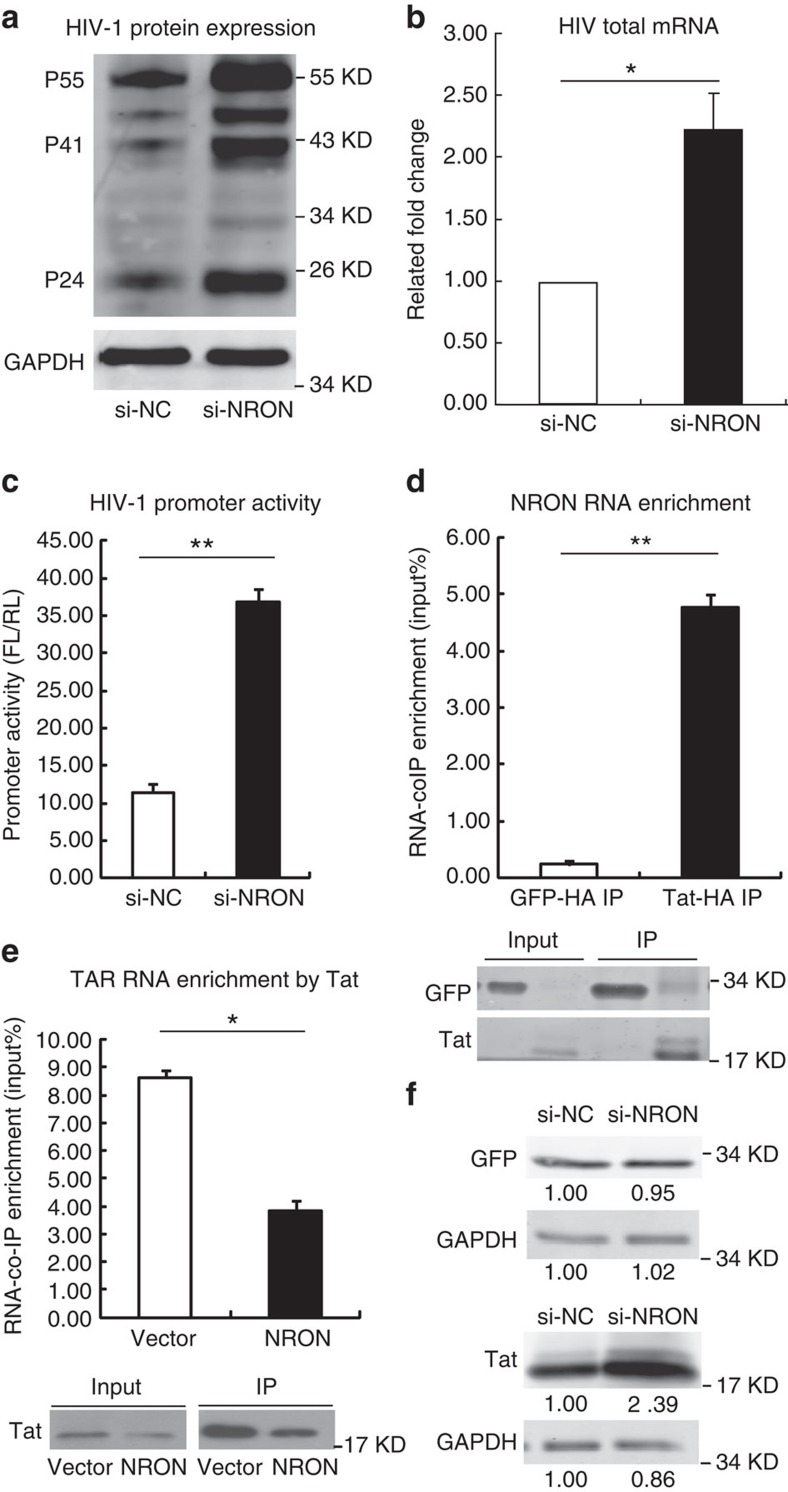
NRON represses HIV-1 transcription by inducing Tat degradation. (**a**) HEK293T cells were co-transfected with pNL4-3-deltaE-EGFP provirus plasmid and siRNAs against NRON or nonspecific control. Gag proteins expression was determined by western blotting with anti-p24 antibody at 72 h after transfection. (**b**) Total HIV-1 mRNAs including spliced and unspliced mRNAs were detected with real-time qRT–PCR in the same HEK293T cells described in **a** (*n*=3). (**c**) HEK293T cells were co-transfected with pHIV-Pro-Luc reporter plasmids, pcDNA3.1-Tat-HA, pRL-CMV (as a transfection normalization reporter) and siRNAs against NRON or nonspecific control. The promoter activity was determined by dual-luciferase reporter assay at 48 h after transfection (*n*=3). (**d**) Overexpressed NRON RNA was co-immunoprecipitated by HA-tagged Tat in TZM-bl cells, and the enriched RNA was determined by real-time qRT–PCR. RNA co-immunoprecipitated by HA-tagged GFP was set as a control (*n*=3), and the precipitated proteins were detected by western blotting. (**e**) The pcDNA3.1-NRON or empty vector was co-transfected with pcDNA3.1-Tat-HA into TZM-bl cells, the TAR-Luc RNA was enriched by RNA co-immunoprecipitation with anti-HA agarose and quantified with real-time qRT–PCR (*n*=3). The precipitated proteins were detected by western blotting. (**f**) Tat and control GFP were detected by western blotting on NRON knockdown in HEK293T cells. Numbers indicated the fold change related to control. Data in **b**–**e** show mean±s.d. (error bars). Results in **a**,**f** represent three independent experiments. *P<0.05, **P<0.01, Student's unpaired *t-*test.

To investigate which stage of HIV-1 replication was likely affected by NRON, we employed the defective HIV-1 provirus plasmid pNL4-3-deltaE-EGFP for transfection assay in HEK293T cells^37^, which also express endogenous NRON RNA at a high level (Supplementary Fig. 2a). The intracellular Gag proteins expression obviously increased when NRON was depleted by siRNAs (Fig. 2a). Conversely, Gag proteins expression decreased when NRON was overexpressed in HeLa cells, which showed a low expression level of endogenous NRON (Supplementary Fig. 2a). Furthermore, the expression level of HIV-1 total mRNA in HEK293T cells showed a significant increase when NRON was knocked down (Fig. 2b), implying that the viral transcription or viral RNA stability could be affected by NRON. As many lncRNAs can regulate gene transcription, we employed a reporter luciferase system for HIV-1 promoter activity to examine whether NRON affected HIV-1 transcription. The HIV-1 promoter activity significantly increased after NRON was depleted in HEK293T cells (Fig. 2c), and was greatly reduced when NRON was overexpressed in TZM-bl cells, which also showed a low expression level of endogenous NRON (Supplementary Fig. 2a,c). To determine whether NRON suppressed the transcription of other viral promoters, we employed reporter luciferase systems for cytomegalovirus (CMV), Moloney murine leukemia virus (MMLV) and Rous sarcoma virus (RSV) promoters. Only slight changes were observed in these promoter reporter systems when NRON was knocked down (Supplementary Fig. 2d–f), indicating that NRON could specifically suppress HIV-1 promoter activity.

### NRON induces Tat protein degradation

On the basis of the above observations, we next sought to identify the targeting site(s) of NRON on HIV-1 promoter. NRON has been reported as a negative regulator of NFAT signalling pathway^18^. As HIV-1 harbours two sets of canonical NFAT-binding site in its 5′ long terminal repeat (LTR), which can enhance the viral transcription^38^,^39^,^40^, we constructed a HIV-1 promoter reporter plasmid with mutations at both these sites. Dual-luciferase reporter assay showed that the activity of NFAT-binding site-mutated HIV-1 promoter still increased on the knockdown of NRON in HEK293T cells (Supplementary Fig. 3a). Further, it has been shown that NFAT signal activity in HEK293T cells was relatively weak without stimulation by phorbol myristate acetate/ionomycin^18^, which is consistent with latently infected resting CD4^+^ T lymphocytes. Therefore, our data indicated that NRON could regulate HIV-1 transcription through a NFAT-independent pathway in our HEK293T cell line monitor system.

The intact HIV-1 5′ LTR promoter contains four functional regions, namely the modulatory region (−454 to −105 nucleotide (nt) relative to the transcription start site), the enhancer region (−104 to −79 nt), the basal promoter (−78 to −1 nt) and TAR element (+1 to + 60 nt)^41^. We constructed mutated HIV-1 promoters with deletions of the modulatory region and the enhancer region, or the TAR element (Supplementary Table 1). Although the promoter activity was significantly decreased because of these mutations, the core HIV-1 promoter with only the basal promoter and the TAR element could still be regulated by NRON (Supplementary Fig. 3b). However, the TAR element-deficient promoter did not exhibit this feature on modification (Supplementary Fig. 3c). It is well known that the TAR element recruits Tat, which then exploits CDK9 and Cyclin-T1 and plays a key role in transcription elongation^42^,^43^,^44^. The specific U-residue-rich bulge (+23 to +25 nt) in the stem of TAR RNA is required for Tat binding, and mutation of the U residues in the bulge will significantly reduce its affinity to Tat^45^,^46^,^47^. When we substituted the U residues in the bulge with G residues in the TAR region to disrupt the Tat–TAR interaction, dual-luciferase assay showed that the mutated promoter was no longer affected by NRON knockdown (Supplementary Fig. 3d). We then investigated whether NRON was still functional in the absence of Tat. We indeed found that, in the absence of Tat-expressing plasmid, the regulation of NRON on HIV-1 promoter activity was no longer significant (Supplementary Fig. 3e). All these data indicated that the Tat–TAR axis was involved in NRON function.

Since Tat is a RNA-binding protein, we initially hypothesized that NRON could bind to Tat protein and disrupt its recruitment to TAR RNA, thus functioning as a competing endogenous RNA^14^. RNA co-immunoprecipitation (RNA co-IP, RIP) and real-time qRT–PCR data showed that the enrichment of NRON by Tat was more than 15-fold increase compared with the green fluorescent protein (GFP) control in NRON-overexpressed TZM-bl cells (Fig. 2d). In addition, Tat could also potently enrich the endogenously expressed NRON in HEK293T cells (Supplementary Fig. 4a), indicating that Tat binds to NRON in an HIV-1 promoter-independent manner. We then confirmed that the association between Tat and TAR-Luc RNA reduced on NRON overexpression in TZM-bl cells (Fig. 2e). Unexpectedly and interestingly, Tat protein abundance, initially detected as the input control by western blotting for RNA co-IP assay, significantly decreased on NRON overexpression in TZM-bl cells (Fig. 2e), while the Tat mRNA level remained unchanged (Supplementary Fig. 4b). Conversely, when NRON was depleted in HEK293T cells, the Tat protein level increased. However, the control GFP protein level showed no significant changes (Fig. 2f). Meanwhile, knockdown of NRON RNA did not affect the protein level of other HIV-1 proteins, such as Nef and Vpr (Supplementary Fig. 4c). Collectively, these data suggested that NRON RNA specifically induced the reduction of Tat at the protein level.

### NRON forms a complex with CUL4B and PSMD11

**Figure 3:**
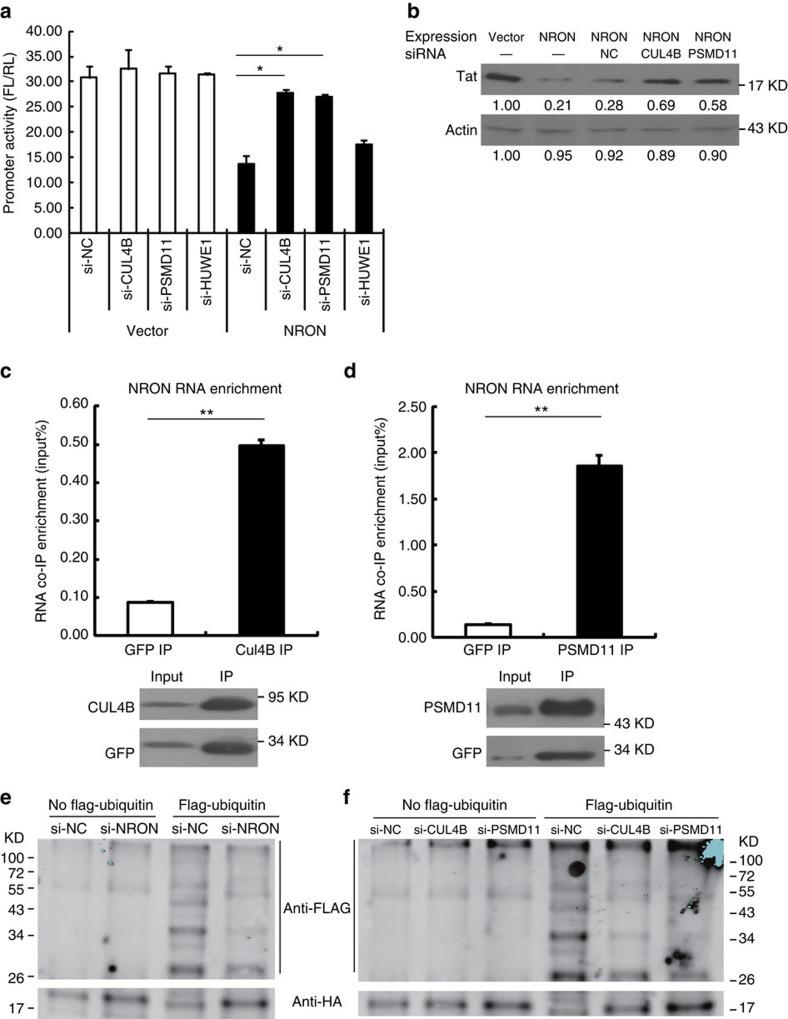
NRON conjugates Tat protein to the ubiquitin/proteasome system and induces its ubiquitination. (**a**) The indicated siRNAs were co-transfected with HIV-1 promoter reporter plasmids into HEK293T cells, and the promoter activities were measured with dual-luciferase reporter assay (*n*=3). (**b**) siRNAs against CUL4B and PSDM11 were co-transfected with pcDNA3.1-Tat-HA and pcDNA3.1-NRON into TZM-bl cells, and the Tat protein levels were detected by western blotting at 48 h post transfection. Numbers indicated the fold change related to control. The endogenous NRON RNA in HEK293T cells was co-immunoprecipitated by ectopically expressed FLAG-tagged CUL4B (**c**) or PSMD11 (**d**) and was quantified using real-time qRT–PCR (*n*=3). *In vitro* ubiquitination assay was performed, and western blotting showed the ubiquitin-labelled Tat when NRON (**e**), CUL4B or PSMD11 (**f**) was knocked down. Data in **a**,**c**,**d** show mean±s.d. (error bars). Results in **b**,**e**,**f** represent three independent experiments. *P<0.05, **P<0.01, Student's unpaired *t-*test.

Because of the findings thus far, we started to notice that three of the reported NRON-binding proteins, CUL4B, PSMD11 and HUWE1 (UREB1), belong to the ubiquitin/proteasome system^18^. We speculated that these proteins were involved in the process of NRON-mediated Tat degradation. To examine this hypothesis, we first investigated the roles of these proteins in HIV-1 transcription by a loss-of-function assay (Supplementary Fig. 4d). The transcription repression of HIV-1 promoter by NRON overexpression in TZM-bl cells was counteracted by CLU4B or PSMD11 knockdown, but not by HUWE1 (Fig. 3a). Correspondingly, the depletion of these proteins remarkably reversed Tat reduction caused by NRON overexpression in TZM-bl cells (Fig. 3b). Meanwhile, NRON RNA could be enriched by CUL4B (Fig. 3c) or PSMD11 (Fig. 3d), indicating that they were associated with NRON RNA robustly. Further, we found that Tat interacted with CUL4B or PSMD11 by co-IP; however, the interaction significantly decreased in the presence of RNase A (Supplementary Fig. 4e). When NRON was knocked down in the HEK293T cells, the interactions between Tat and CUL4B or PSMD11 were significantly diminished (Supplementary Fig. 4f). Moreover, the *in vitro* ubiquitination assay and the subsequent western blotting detection showed that Tat ubiquitination was significantly decreased when NRON was depleted (Fig. 3e). The same result was observed when CUL4B or PSMD11 was knocked down (Fig. 3f). Collectively, these data suggested that NRON mediated Tat degradation by bridging Tat and the components of ubiquitin/proteasome, and by facilitating the ubiquitination of Tat protein.

### NRON participates in maintaining HIV-1 latency

**Figure 4:**
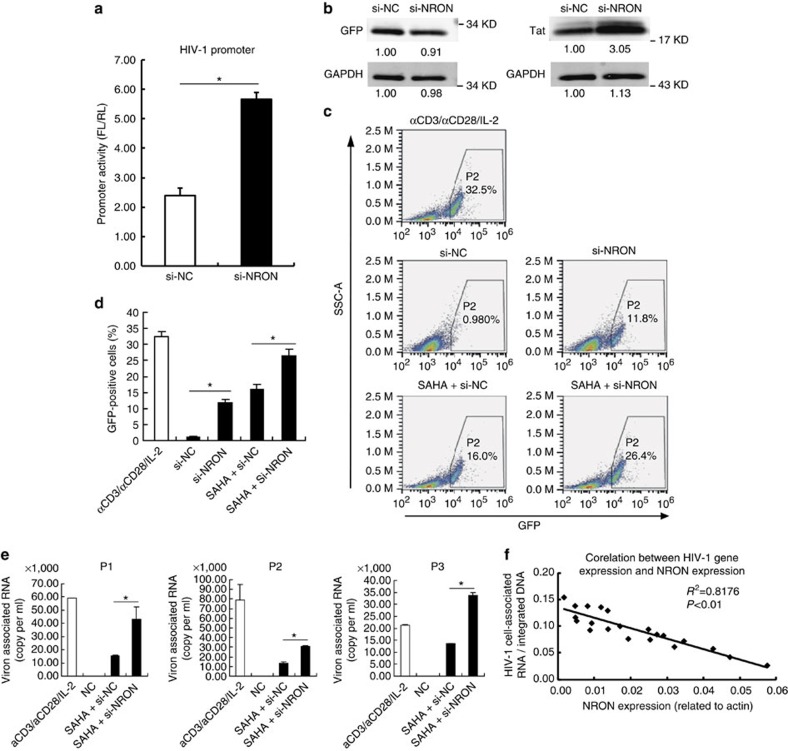
Depletion of NRON reactivates HIV-1 viruses in latently infected CD4^+^ T lymphocytes. (**a**) Primary resting CD4^+^ T lymphocytes were nucleofected with HIV-1 promoter reporter system plasmids, pcDNA3.1-Tat-HA and siRNAs against NRON or nonspecific control. The promoter activity was determined with dual-luciferase reporter assay at 48 h after transfection (*n*=3). (**b**) Tat and control GFP were detected by western blotting on NRON knockdown in nucleofected primary resting CD4^+^ T lymphocytes. Numbers indicated the fold change related to the control. (**c**) The latently infected cells were transfected with NRON siRNAs or nonspecific control, or were transfected with siRNAs in combination with the treatment of SAHA, and detected by FACS at 48–72 h post transfection. The GFP+ ratio indicated the reactivation level (**d**; *n*=3). (**e**) Resting CD4^+^ T lymphocytes isolated from HIV-1-infected individuals on suppressive cART were transfected with siRNAs in combination with the treatment of SAHA. After 48 h, HIV-1 virion-associated RNAs in the supernatants were isolated and detected with real-time qRT–PCR (*n*=3). (**f**) The intracellular HIV-1 RNA and NRON RNA expression levels were detected in resting CD4^+^ T lymphocytes isolated from HIV-1-infected individuals on suppressive cART (*n*=20), and the correlation between the HIV-1 RNA and NRON RNA levels was shown. The simple linear regression analysis was performed and linear regression line was shown. Data in **a**,**d**,**e** show mean±s.d. (error bars). Results in **b** represent three independent experiments. *P<0.05, Student's unpaired *t-*test.

Since NRON showed higher expression level in resting CD4^+^ T lymphocytes than in activated cells, we sought to determine whether this lncRNA functions in HIV-1 latent infection. Initially, we confirmed whether NRON could regulate HIV-1 transcription in primary resting CD4^+^ T lymphocytes. HIV-1 promoter reporter system plasmids, Tat-expressing plasmids, control siRNA or NRON siRNA were nucleofected into resting CD4^+^ T lymphocytes by electroporation. Dual-luciferase reporter assay showed that the activity of HIV-1 promoter significantly increased on NRON knockdown (Fig. 4a), which suggested that NRON RNA suppressed HIV-1 transcription in resting CD4^+^ T lymphocytes. We then examined the effect on Tat protein in resting NRON-depleted CD4^+^ T lymphocytes. Western blotting showed that the Tat protein level significantly increased on NRON knockdown (Fig. 4b). These data indicated that NRON RNA could suppress HIV-1 transcription by inducing Tat protein degradation in resting CD4^+^ T lymphocytes, which were the major population of HIV-1-latently infected cells.

Further, we generated a modified primary CD4^+^ T lymphocyte-based latency model^48^,^49^. A *bcl-2* open reading frame (ORF) was properly inserted into the *nef* ORF in the defective HIV-1 provirus plasmid pNL4-3-deltaE-EGFP to express Bcl-2 protein for promoting cell survival (Supplementary Fig. 5a,b). The resulting HIV/VSV (vesicular stomatitis virus)-pseudotyped viruses were used to infect the activated human primary CD4^+^ T lymphocytes. The GFP+ cells were enriched and expanded, and then cultured for ∼1 month to allow the cells to convert to resting status (Supplementary Fig. 5d,e). When we transfected the cells with NRON-specific siRNAs, the viruses in the latently infected cells were significantly reactivated (Fig. 4c,d and Supplementary Fig. 5f). Furthermore, after transfection with NRON-specific siRNAs supplemented with the treatment of suberoylanilide hydroxamic acid (SAHA), a well-known histone deacetylase (HDAC) inhibitor and latency-reversing agent (LRA)^50^, the reactivation level was much higher than that treated with nonspecific siRNA in combination with SAHA (Fig. 4c,d). Moreover, we examined the effect of knockdown of NRON upon HIV-1 latency in resting CD4^+^ T lymphocytes directly isolated from HIV-1-infected individuals receiving suppressive cART. Alu-PCR was employed to confirm that the cells harboured proviral HIV-1 DNA (Supplementary Fig. 5g)^34^. After transfection of the resting cells with NRON-specific siRNAs in combination with SAHA treatment, the virion-associated RNA in the culture supernatant was significantly higher than that treated with nonspecific siRNA in combination with SAHA (Fig. 4e and Supplementary Fig. 5h). No significant change was observed by NRON knockdown only (Supplementary Fig. 5i), which may be because of the fact that the proviruses were more difficult to be reactivated from the primary resting CD4^+^ T lymphocyte directly isolated from HIV-1-infected individuals receiving suppressive cART than that from the HIV-1-latently infected primary CD4^+^ T lymphocytes, which were experimentally generated.

We then directly isolated resting CD4^+^ T lymphocytes from HIV-1-infected individuals receiving suppressive cART, and detected the intracellular HIV-1 proviral DNA, the expression level of HIV-1 RNA and NRON RNA. We found that the intracellular HIV-1 RNA expression, after being normalized with the cell-associated viral DNA, was inversely correlated with the NRON RNA expression level (Fig. 4f). Collectively, these data indicated that NRON contributed to HIV-1-latent infection. The abundance of NRON in resting CD4^+^ T lymphocytes efficiently blocks the expression of viral proteins and could, therefore, allow the infected cells to evade from the immune surveillance *in vivo*.

## Discussion

Because of their essential regulatory roles in various cellular molecular networks, it is easy to understand why lncRNAs have been implicated in viral infection and immune regulation. A recent report showed that the lncRNA NEAT1 possessed an unspecific effect on the exporting of many mRNAs including HIV-1 RNA^51^. The NRON lncRNA was previously implicated in the severity of inflammatory bowel disease through its inhibitory effects on NFAT signalling^18^,^52^. As HIV-1 harbours NFAT-binding sites in its 5′ LTR promoter region^38^,^39^,^40^, it is highly likely that NRON may suppress HIV-1 replication in activated primary CD4^+^ T lymphocytes through inhibiting NFAT signal, which was described in a recent report by others^53^. However, in latently infected resting CD4^+^ T lymphocytes, NFAT signal activity is relatively weak without stimulation, which is consistent with our HEK293T and HeLa cell line monitor system^18^,^28^. In these systems, the mutation of NFAT-binding sites in the promoter did not affect the inhibitory effect of NRON on HIV-1 transcription (Supplementary Fig. 3a). In addition, knockdown of NRON could significantly enhance the viral production from latently infected resting CD4^+^ T lymphocytes without reactivating the cells (Fig. 4). These data support our hypothesis that NRON RNA could potently inhibit HIV-1 transcription and play a role in maintaining HIV-1 latency in an NFAT-independent mechanism in resting cells.

Our work further indicates that, by forming a complex with CUL4B and PSMD11, NRON lncRNA recruits the essential HIV-1 regulatory protein Tat to the ubiquitin/proteasome system and induces its protein degradation. In this scenario, NRON functions as an adaptor between the ubiquitin/protease system and its target protein. We have found that the induced degradation of viral protein by NRON is of specificity, as NRON does not affect the stability of Nef and Vpr proteins. Tat contains several lysine amino-acid residues, and the ubiquitination of K71 enhances its transactivation activity, but not degradation of the protein^54^. The effect of NRON complex-induced Tat ubiquitination therefore could be due to ubiquitination at other lysine residues. More detailed studies are needed to identify the entire molecular mechanism. Further, it is notable that NRON in human has one spliced transcript, whereas three spliced transcripts of NRON have been found in mice. It remains to be determined whether NRON also induced the degradation of other proteins in human or mouse cells. Conversely, the specificity of the NRON–CUL4B/PSMD11 interaction and whether other components of the ubiquitin/proteasome system are involved in this process remains to be determined in future studies. As lncRNA HOTAIR could also be an adaptor between the ubiquitin system and its target proteins Ataxin-1 and Snurportin-1 (ref. 55), it may be interesting to systematically investigate whether other lncRNAs could mediate the specific degradation of their binding proteins and the molecular mechanisms underlying these processes.

Because of latency, productive viral replication does not occur in resting primary CD4^+^ T lymphocytes of HIV-1-infected individuals receiving suppressive cART. However, replication-competent proviral DNA and multiply spliced or unspliced viral RNA can easily be found in these cells^56^,^57^,^58^,^59^,^60^. The deficiency of Tat protein is the direct consequence of several latency mechanisms such as the deficiency of transcriptional factors such as NF-kB or NFAT, condensed chromatin structure and epigenetic regulation, as well as the enrichment of suppressive cellular microRNAs^27^,^28^,^30^,^31^,^32^,^33^,^34^. It has been well known that the Tat–TAR interaction leads to the recruitment of the pTEFb complex including CDK9/cyclin-T1, which hyperphosphorylates of the C-terminal domain of RNA polymerase II and significantly increases transcription efficiency^42^,^43^,^44^. Recent studies have demonstrated that this positive-feedback regulation strongly controls HIV-1 latency, and Tat induction could robustly activate latently infected viruses^61^. Our work suggests that, by specifically inducing the degradation of Tat protein, NRON at high concentration in the resting CD4^+^ T lymphocytes prevents the accumulation of Tat and therefore acts as a barrier for the transcriptional activation of proviruses. The endogenous NRON expression level in the resting CD4^+^ T lymphocytes of HIV-1-infected individuals is inversely correlated with the intracellular viral RNA expression level, further supporting this hypothesis. Taken together, we propose a novel mechanism contributing to HIV-1 latency maintenance and also a new target for the development of LRAs^62^.

## Methods

### Ethics statement

This research was approved by the Ethics Review Board of Sun Yat-Sen University and Guangzhou 8th People's Hospital. Written informed consent was provided by all study participants.

### Plasmids and constructs

HIV-1 infectious clone pNL4-3 and its derivative pNL4-3-deltaE-EGFP were obtained through the NIH AIDS Reagent Program, Division of AIDS, NIAID, NIH (refs 37, 63). The pHIV-Pro-Luc reporter plasmid was constructed by replacing the CMV promoter of the *luciferase* gene in the pMIR-REPORT Luciferase vector (Ambion) with the 5′ LTR sequence of HIV-1_NL4-3_. Mutations or deletions were introduced into the promoter by PCR-based strategies, and the primer pairs were listed in Supplementary Table 1. The *bcl-2* gene was chemically synthesized and inserted into the *nef* region of pNL4-3-deltaE-EGFP provirus plasmid at the XhoI cutting site. Its reading frame is consistent with the *nef* ORF.

The two exons of HIV-1 full-length *tat* with an haemagglutinin (HA) epitope tag at its exon-2 3′ terminus were PCR-amplified from pNL4-3 clone. After connection of two fragments with overlapping PCR, it was inserted into the pcDNA3.1 vector. Human full-length 2,735-bp *NRON* was amplified using PCR with total cDNA of human peripheral blood mononuclear cells (PBMCs) as the template. Human *CUL4B* and *PSMD11* with a FLAG epitope tag sequence at their 3′ terminus was amplified using PCR with total cDNA of human PBMCs as the template. *NRON*, tagged *CUL4B*, or *PSMD11* were then inserted into the pcDNA3.1 vector. All constructs were verified by DNA sequencing. The *gfp* coding sequence (CDS) was tagged with HA or FLAG tag at the C terminus and was constructed into the pcDNA3.1 vector^64^. The N-terminus FLAG-tagged human ubiquitin B was constructed into the pcDNA3.1 vector^65^. The renilla luciferase-expressing plasmid pRL-CMV was obtained from Promega as a transfection normalization reporter for dual-luciferase reporter assay.

### Cells and transfection

TZM-bl cells, which harbour an HIV-1 promoter-driven luciferase gene, were obtained from AIDS Reference Reagent Program, NIH. Human HEK293T (American Type Culture Collection (ATCC)), HeLa (ATCC) and TZM-bl cells were maintained in DMEM (Hyclone) supplemented with 10% fetal bovine serum (Invitrogen), 100 units ml^−1^ of penicillin and 100 μg ml^−1^ of streptomycin at 37 °C. All the cell lines had been tested for mycoplasma using a PCR assay and were mycoplasma-free. The HEK293T, HeLa and TZM-bl cells were transfected using Lipofectamine 2000 (Invitrogen) for plasmids and siRNAs by following the manufacturer's instructions. The cells were collected at 48 h post transfection for dual-luciferase reporter assays and protein detection. Human primary CD4^+^ T lymphocytes were nucleofected with Amaxa Nucleofector using the Amaxa Human T Cell Nucleofector Kit according to the manufacturer's instructions.

### Purification of human primary CD4^+^ T lymphocytes

The PBMCs were isolated from healthy human donors through Ficoll gradient centrifugation, followed by culturing in the conditioned RPMI 1640 medium. Human primary CD4^+^ T lymphocytes were then purified with a human CD4^+^ T-cell isolation kit according to the manufacturer's instructions (BD Biosciences). The isolated human primary CD4^+^ T lymphocytes were then maintained in the conditioned RPMI 1640 medium (Hyclone) and stimulated with phytohaemagglutinin (5 ng ml^−1^, Roche Applied Science) and interleukin-2 (IL-2, 10 ng ml^−1^, R&D Systems), or anti-CD3 antibody (1 μg ml^−1^, BD Biosciences), anti-CD28 antibody (1 μg ml^−1^, BD Biosciences) and IL-2 (10 ng ml^−1^, R&D Systems) for 48 h. Then, the cells were washed three times with PBS buffer, and were cultured in the presence of IL-2 (10 ng ml^−1^).

### HIV-1 infection

HEK293T cells at 3 × 10^6^ per dish were plated on 100-mm cell culture dish. Twenty-four hours later, the cells were transfected with 10 μg of pNL4-3 plasmids with Lipofectamine 2000 (Invitrogen) according to the manufacturer's instructions. Cell supernatants were harvested at 48 h post transfection and were stored at −80 °C. To normalize viral inputs, the amount of p24 was measured using an HIV-1 p24 ELISA kit according to the manufacturer's instructions (Clonetech). The activated primary CD4^+^ T lymphocytes were seeded into 12-well cell-culture plates (2 × 10^6 ^ml^−1^, 1 ml per well) and were transfected with 200 pmol lncRNA-specific siRNAs or nonspecific control with Lipofectamine RNAiMAX (Invitrogen) according to the manufacturer's instructions. Eight to twelve hours later, the cells were infected with the equivalent of 10 ng HIV-1 p24 antigen per well for 3 h at 37 °C. Then, the supernatants were removed and the cells were washed three times with fresh PBS buffer. The cells were maintained in the conditioned RPMI 1640 medium supplemented with IL-2 (10 ng ml^−1^). The culture supernatants were collected on day 7 post infection. For the time-course study of the viral replication, at 7 days post infection, the cells were transfected with NRON siRNAs or nonspecific control for the second time. The culture supernatants were collected at 12 h (day 0), day 3, day 7, day 10 or day 14 post infection. All of the culture supernatants were detected using the HIV-1 p24 ELISA kit according to the manufacturer's instructions (Clonetech).

### The chemical synthesis of siRNAs

Two or three specific siRNAs for one target lncRNA or protein were pooled together to reduce off-target effects. The chemically synthesized siRNAs and nonspecific control were purchased from RiboBio (Guangzhou). The target sequences of siRNAs were listed in Supplementary Table 2.

### Luciferase assay

Twenty-four hours before transfection, HEK293T cells (2 × 10^4^ cells per well) or TZM-bl cells (2.5 × 10^4^ cells per well) were seeded into 48-well plates. To perform the reporter assay for wild-type or mutated HIV-1 promoter activities, 2 ng of reporter plasmids, 0.2 ng of pRL-CMV and 2 ng of pcDNA3.1-Tat-HA were co-transfected with 30 pmol of siRNAs or nonspecific control into HEK293T cells. In all, 1 ng of pRL-CMV, 5 ng of pcDNA3.1-Tat-HA, 40 ng of pcDNA3.1-NRON and/or 30 pmol of siRNAs were co-transfected into TZM-bl cells. To perform the reporter assay for CMV, MMLV or RSV promoter activities, 2 ng of pMIR-Reporter or 10 ng of MMLV or RSV promoter reporter plasmids were transfected with 30 pmol of siRNAs or nonspecific control into HEK293T cells. Human primary CD4^+^ T lymphocytes (3–5 × 10^6^ cells per well) from healthy donors were nucleofected with 2 μg of HIV-1 promoter reporter plasmid, 200 ng of pRL-CMV, 2 μg of pcDNA3.1-Tat-HA and 100 pmol of siRNAs. Dual-luciferase reporter assay was performed at 48 h post transfection using the Promega Dual-Luciferase Reporter Assay System according to the manufacturer's instructions^66^,^67^.

### RNA isolation and real-time qRT–PCR

RNA was isolated with TRIZOL reagent according to the manufacturer's instructions (Ambion), and was treated with RQ-1 DNase (Promega) before reverse transcription. Reverse transcription reactions were performed with the PrimeScript RT reagent Kit (TaKaRa). Quantitative PCR was performed with the SYBR Premix ExTaq Kit (TaKaRa) on a CFX96 Real-Time System (Bio-Rad) by following the manufacturer's instructions. Primers for real-time qRT–PCR were listed in Supplementary Table 3. Human glyceraldehyde-3-phosphate dehydrogenase (GAPDH) or β-actin mRNA was measured as endogenous controls. For RIP–qPCRs, the amount of a target RNA was normalized to 10% total input sample RNA level in each RIP sample and was represented as percentage relative to input sample using the delta Ct method^68^.

### Co-IP and western blotting

HEK293T cells at 1.2 × 10^6^ per dish were plated onto 60-mm cell culture dish. Twenty-four hours later, the cells were transfected with 1 μg of pcDNA3.1-Tat-HA and/or 0.8 μg of pcDNA3.1-CUL4B-FLAG or pcDNA3.1-PSMD11-FLAG. The cells were co-transfected with 400 pmol of specific or control siRNAs when needed. Alternatively, 1.5 × 10^6^ TZM-bl cells were plated onto 60-mm cell culture dish. Twenty-four hours later, the cells were transfected with 1 μg of pcDNA3.1-NRON, 1.5 μg of pcDNA3.1-Tat-HA and/or 1 μg of pcDNA3.1-CUL4B-FLAG or pcDNA3.1-PSMD11-FLAG. Cells were co-transfected with 400 pmol of specific or control siRNAs when needed. At 48 h post transfection, cells were collected and disrupted with RIPA lysis buffer (150 mM NaCl, 50 mM Tris-HCl (pH 7.5), 1 mM EDTA, 1% NP40 and 0.5% Triton X-100) containing protease inhibitor cocktail (Sigma) and RNaseOut (Invitrogen) for 30 min on ice. The cell lysates were clarified with centrifugation at 12,000 *g* for 10 min at 4 °C. The supernatants were pre-cleared with agarose beads, and then mixed with anti-HA beads (Sigma A2095) or anti-FLAG beads (Sigma F2426) and incubated at 4 °C for 4 h to overnight. The beads were washed five times with cold lysis buffer at 4 °C. RNase A was added to wash buffer (20 ug ml^−1^) when needed. RNA isolation followed by real-time qRT–PCR, or western blotting, was then performed^67^. The anti-HA antibody (1:2,000 diluted, mouse monoclonal, MBL International Corporation, M180-3), anti-FALG antibody (1:1,000 diluted, rabbit polyclonal, MBL International Corporation, PM020), anti-β-actin antibody (1:1,000 diluted, rabbit polyclonal, CST #4967), anti-GAPDH antibody (1:5,000 diluted, rabbit polyclonal, Proteintech Group, 10494-1-AP) or anti-p24 antibody (1:500 diluted)^64^ were used as the primary antibodies. Images were analysed using the Quantity One Software (Bio-Rad). The uncropped scans of the most important blots were supplied in Supplementary Fig. 6.

### *In vitro* ubiquitin assay

HEK293T cells at 1.2 × 10^6^ per dish were plated onto 60-mm cell culture dish. Twenty-four hours later, the cells were transfected with 1 μg of pcDNA3.1-Tat-HA, 2 μg of pcDNA3.1-Flag-Ub or empty pcDNA3.1 vector, and 400 pmol of specific or control siRNAs. MG132 (5 μM) was added into the cultures 12 h before the cells were harvested. At 48 h post transfection, cells were collected and disrupted by lysis buffer for anti-HA immunoprecipitation. Western blot analysis was then performed with anti-HA or anti-FLAG primary antibodies.

### Northern blotting

For NRON lncRNA detection, 20 μg of total RNA was separated by 1.2% formaldehyde-denature agarose gel, and then transferred to Amersham Hybond-N^+^ membrane (GE Healthcare) and ultraviolet-crosslinked RNA to the membrane. The membrane was prehybridized with salmon sperm DNA (100 μg ml^−1^, Sigma), and was hybridized with NRON and Actin-specific probes. Then, the membrane was washed three times, dried, exposed on a phosphorimager screen (GE Healthcare) and scanned on a laser scanner (GE Healthcare)^69^. The reverse primers of NRON and Actin for real-time qRT–PCR were used as oligonucleotide probes, and were labelled with α-^32^P (PerkinElmer) by T4 polynucleotide kinase (NEB).

### HIV *in vitro* latency model

The HIV-1/VSV-pseudotyped viruses were packaged in HEK293T cells by co-transfecting pNL4-3-deltaE-EGFP-Bcl-2 and pVSV-G. The activated primary CD4^+^ T lymphocytes isolated from healthy individuals were infected with the pseudotyped viruses, and the GFP-positive cells were sorted out by FACS and expanded by culturing in the conditioned RPMI 1640 medium containing anti-CD3 antibody (1 μg ml^−1^, BD Biosciences), anti-CD28 antibody (1 μg ml^−1^, BD Biosciences) and IL-2 (10 ng ml^−1^, R&D Systems) for 7 days. The cells were cultured for ∼4 weeks with low concentration of IL-2 (2 ng ml^−1^ R&D Systems), and then the GFP-negative cells were enriched by FACS and subjected to activation by various reagents.

### HIV-1-infected individual samples

The HIV-1-infected individuals with the blood plasma viral RNA less than 20 copies per ml and the number of CD4^+^ lymphocytes higher than 200 μl^−1^ were recruited for our study. The PBMCs were isolated through Ficoll gradient centrifugation, followed by culture in the conditioned RPMI 1640 medium. For NRON knockdown assay, the integrated HIV-1 proviruses in the resting CD4^+^ T lymphocytes were confirmed with Alu-PCR^34^. The resting CD4^+^ T lymphocytes were transfected with siRNAs (200 nM) and treated in combination with SAHA (300 nM), or with anti-CD3 antibody, anti-CD28 antibody and IL-2 as positive controls. Viral RNAs in culture supernatant were isolated and detected by real-time qRT–PCR at 48 h after transfection^34^. For intracellular RNA expression and proviral DNA detection, total RNA and DNA were directly isolated from the primary resting CD4^+^ T lymphocytes. HIV-1 viral RNA and NRON RNA expression levels were detected with real-time qRT–PCR, and then normalized to the endogenous control β-actin mRNA using the delta Ct method^34^. The integrated proviral DNA levels were detected using real-time PCR with a primer set targeting Gag ORF^34^,^56^, and then normalized to β-actin DNA using the delta Ct method^68^.

### Statistical analysis

Results of the experiments were presented as mean±s.d. (error bars). Student's unpaired *t-*test was used to determine significance. *P<0.05 and **P<0.01 indicated significant difference. Correlation analysis was determined using a simple linear regression analysis in the SPSS software.

### Data availability

The data that support the findings of this study are available from the corresponding author upon request.

## Additional information

**How to cite this article:** Li, J. *et al.* Long noncoding RNA NRON contributes to HIV-1 latency by specifically inducing tat protein degradation. *Nat. Commun.* 7:11730 doi: 10.1038/ncomms11730 (2016).

## Supplementary Material

Supplementary Information Supplementary Figures 1-6 and Supplementary Tables 1-3
