# Supplementary material for: Long noncoding RNA NRON contributes to HIV-1 latency by specifically inducing tat protein degradation
Source: Nat Commun. 2016 Jun 13;7:11730. doi: 10.1038/ncomms11730 (PMC4909936; doi:10.1038/ncomms11730)
Supplement: Supplementary Information — Supplementary Figures 1-6 and Supplementary Tables 1-3 [file ncomms11730-s1.pdf]

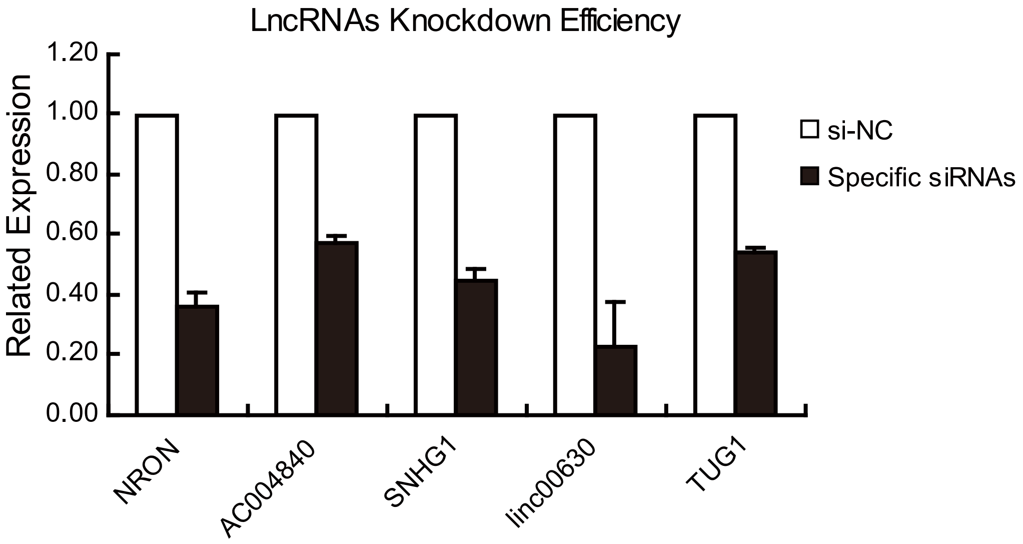

Supplementary Figure 1

Supplementary Figure 1 Knockdown of lncRNAs by siRNAs pools. The lncRNAs knockdown efficiency was detected by real-time qRT-PCR in the activated primary CD4+ T lymphocytes (n=3). The results show mean ± S.D. (error bars).

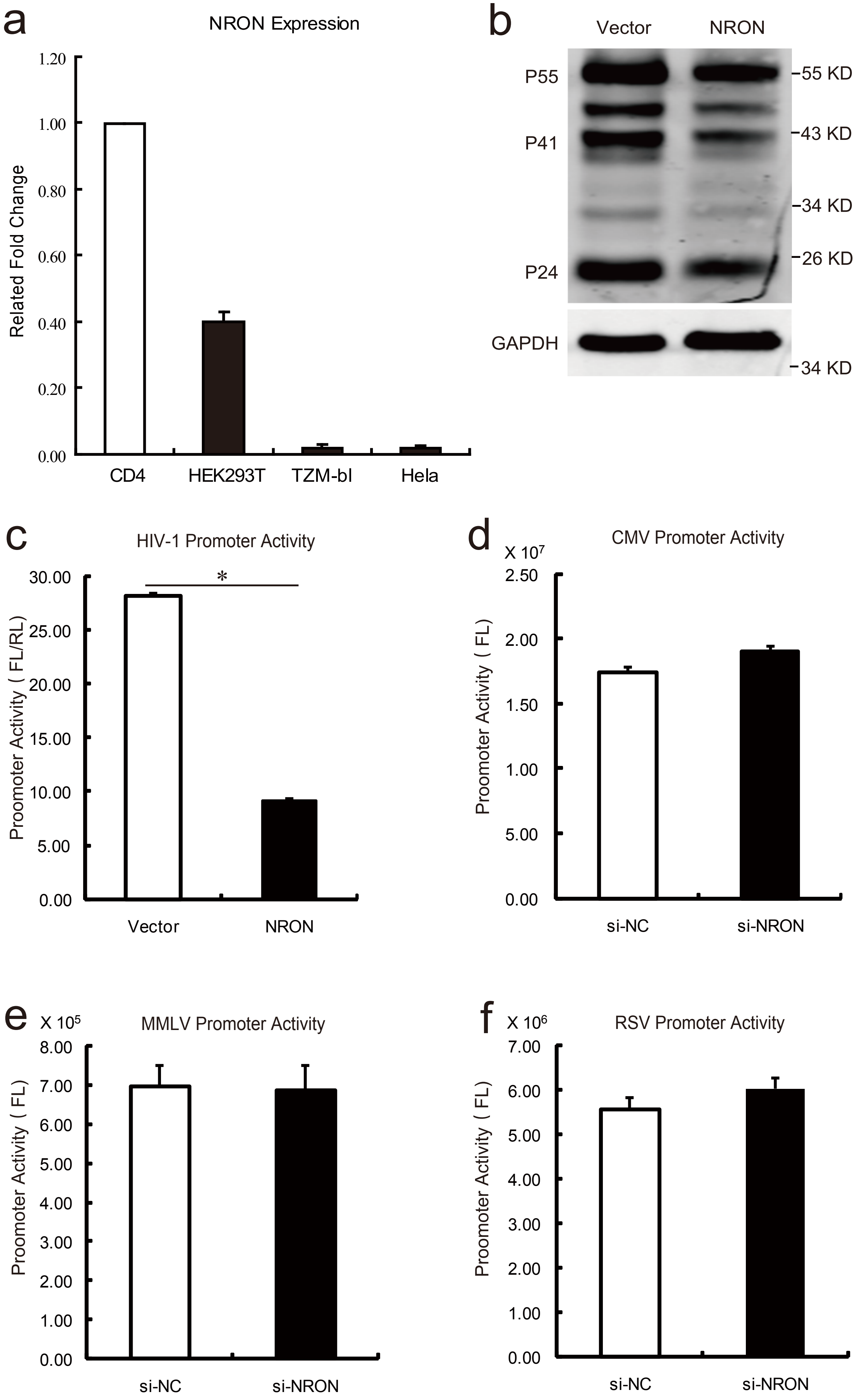

Supplementary Figure 2

Supplementary Figure 2 NRON specifically inhibits HIV-1 promoter activity. (**a**) NRON expression level in activated primary $CD4^+$ T lymphocytes, HEK293T, TZM-bl and HeLa cells was detected by real-time qRT-PCR. The expression of NRON in activated primary $CD4^+$ T lymphocytes was normalized as 1 (n=3). (**b**) HeLa cells were co-transfected with pNL4-3-deltaE-EGFP plasmids and NRON-expressing plasmid pcDNA3.1-NRON or empty vector control. Gag proteins expression was determined by western blotting with anti-p24 primary antibody at 72 h after transfection. (**c**) pcDNA3.1-Tat-HA, pRL-CMV and pcDNA3.1-NRON, or empty vector were co-transfected into TZM-bl cells, and at 48 h after transfection the integrated HIV-1 promoter activity was determined by dual-luciferase assay (n=3). (**d-f**) CMV, MMLV, or RSV promoter activities were examined upon NRON knockdown by luciferase reporter assay (n=3). Data in **a**, **c-f** show mean ± S.D. (error bars). Results in **b** represent three independent experiments. * $P<0.05$, Student's unpaired *t* test.

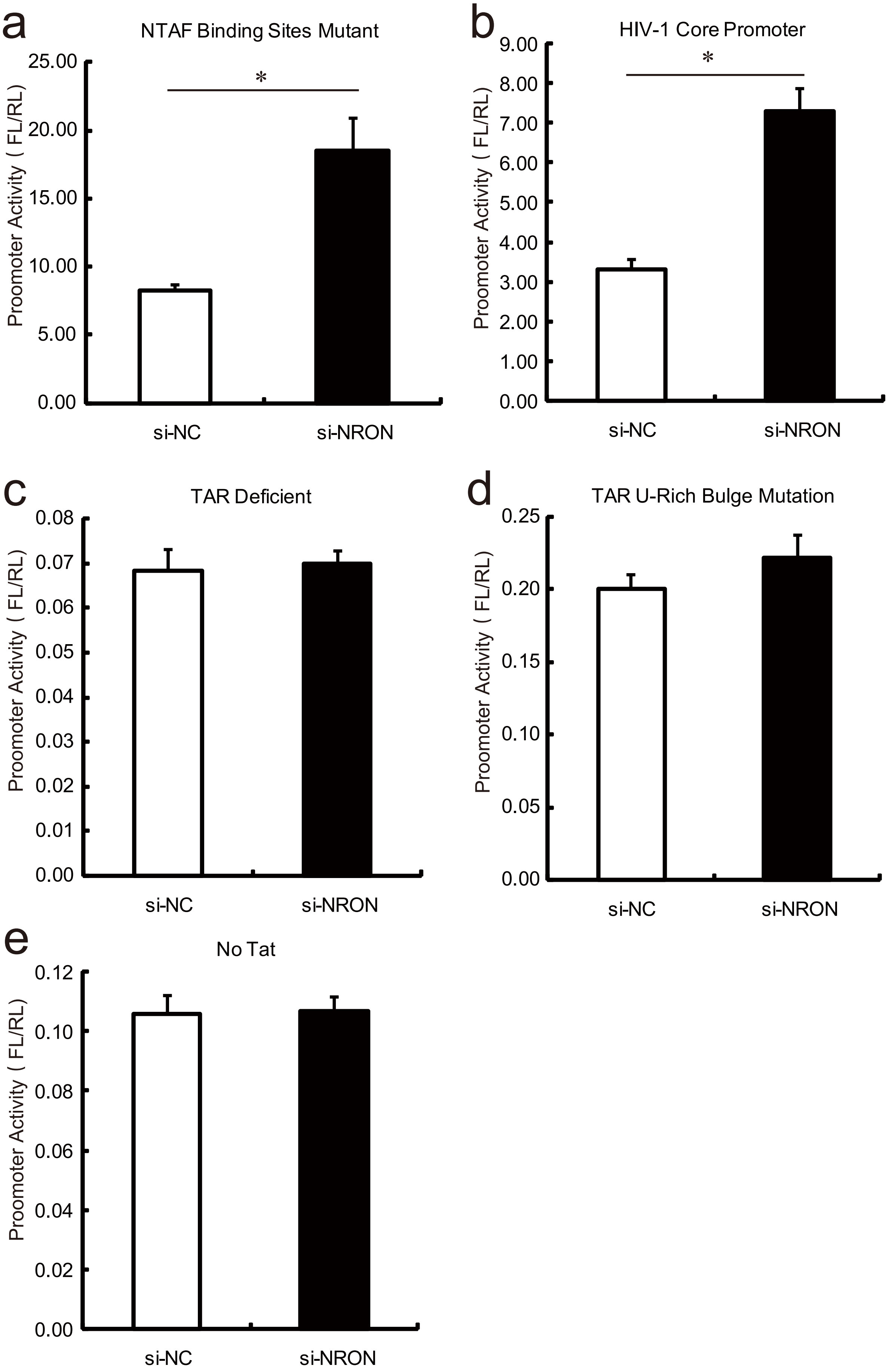

Supplementary Figure 3

Supplementary Figure 3 NRON targets the HIV-1 Tat/TAR axis. The activities of HIV-1 NFAT binding sites mutated promoter (**a**) or core promoters (**b**) were determined by dual-luciferase reporter assay in HEK293T cells upon NRON depletion. The activities of TAR element deficient (**c**) or Tat binding U-rich bulge mutated (**d**) HIV-1 promoters were measured by dual-luciferase reporter assay. (**e**) HIV-1 promoter activity was determined upon NRON knockdown without co-transfection of the Tat-expressing plasmid pcDNA3.1-Tat-HA in HEK293T cells. The results show mean ±S.D. (error bars) from triplicate samples. * $P<0.05$, Student's unpaired *t* test.

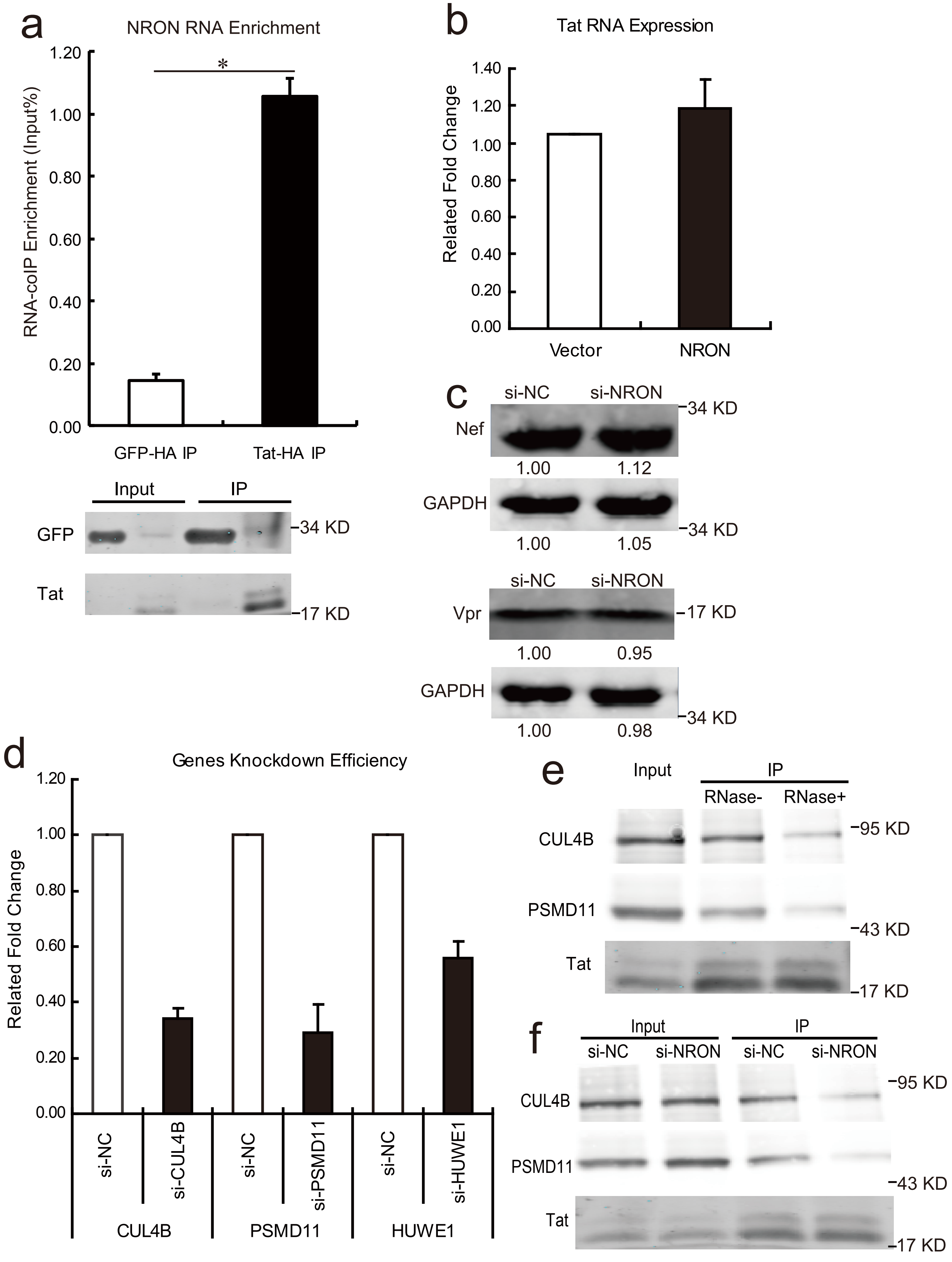

Supplementary Figure 4

Supplementary Figure 4 NRON recruits Tat to the ubiquitin/proteasome system. (**a**) Endogenous RNA was co-immunoprecipitated by Tat in HEK293T cells, the enriched NRON RNA was determined by real-time qRT-PCR (n=3), and the precipitated proteins were detected by western blotting. (**b**) The pcDNA3.1-NRON or empty vector were co-transfected with pcDNA3.1-Tat-HA into TZM-bl cells, and Tat RNA level was detected by real-time qRT-PCR (n=3). (**c**) Nef and Vpr were detected by western blotting upon NRON knockdown in HEK293T cells. Numbers indicated the fold change related to control. (**d**) Knockdown efficiency of CUL4B, PSMD11, and HUWE1 was confirmed by real-time qRT-PCR (n=3). (**e**) The ectopic expressed CUL4B and PSMD11 in HEK293T cells were co-immunoprecipitated by Tat. The precipitates were washed with RNase-free or RNase A containing washing buffer and the enriched CUL4B or PSMD11 were detected by western blotting. (**f**) SiRNAs against NRON or non-specific control and pCDNA3.1-Tat-HA were co-transfected into Flag-tagged CUL4B or PSMD11-expressing HEK293T cells, and then CUL4B or PSMD11 were co-immunoprecipitated by Tat and detected by western blotting. Data in **a**, **b** and **d** show mean ± S.D. (error bars). Results in **c**, **e** and **f** represent three independent experiments. * $P<0.05$, Student's unpaired *t* test.

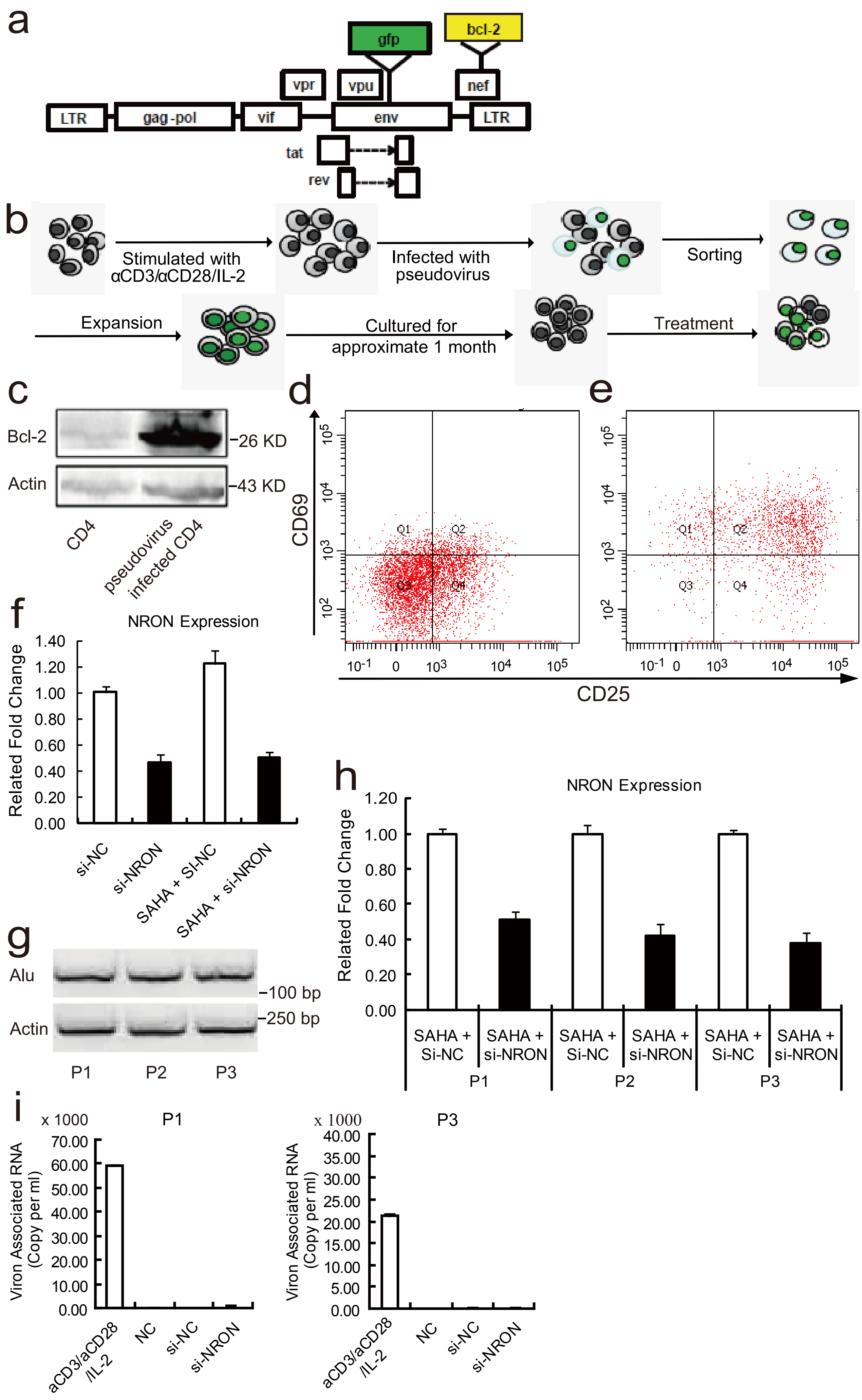

Supplementary Figure 5

Supplementary Figure 5 HIV-1 latency models. (**a**) The *bcl-2* gene was inserted into the *nef* region of pNL4-3-deltaE-EGFP. (**b**) Schematic representation of generating the modified *in vitro* latency model with primary $CD4^+$ T lymphocytes. (**c**) Western blotting detection of Bcl-2 in the activated human primary $CD4^+$ T lymphocytes infected by pNL4-3-deltaE-EGFP-Bcl-2 pseudotyped viruses. After cultured for approximately 1 month (**d**), or then reactivated by anti-CD3 and anti-CD28 antibodies stimulation for 48 h (**e**), the status of the pseudotyped viruses infected primary $CD4^+$ T lymphocytes were confirmed by FACS. NRON knockdown efficiency was detected in the primary $CD4^+$ T lymphocytes *in vitro* latency model (**f**) and resting $CD4^+$ T lymphocytes isolated from HIV-1 infected individuals (**h**). (**g**) Alu-PCR confirmation of the integrated HIV-1 provirus in the resting $CD4^+$ T lymphocytes isolated from HIV-1 infected individuals receiving suppressive cART. (**i**) Resting $CD4^+$ T lymphocytes isolated from HIV-1 infected individuals on suppressive cART were transfected with control or NRON-specific siRNAs. After 48 h, HIV-1 virion associated RNAs in the supernatants were isolated and detected by real-time qRT-PCR. Results in **c**, **d**, **e** and **g** represent three independent experiments. Data in **f**, **h** and **i** show mean ± S.D. (error bars).

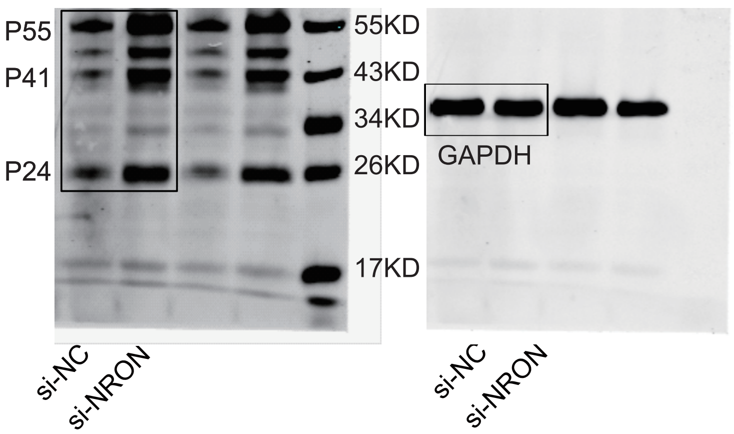

Fig 2a

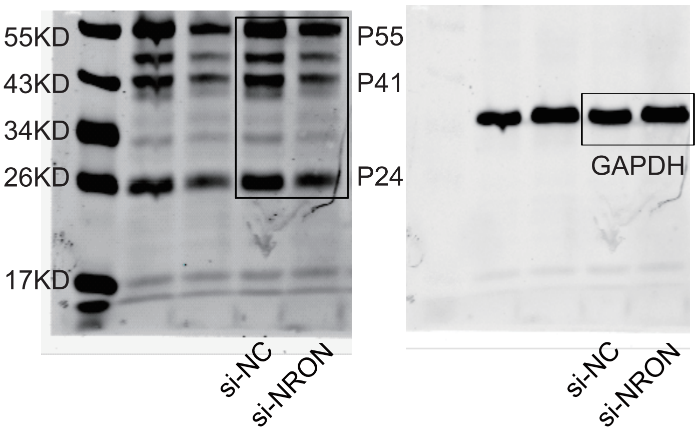

Supplementary Fig 2b

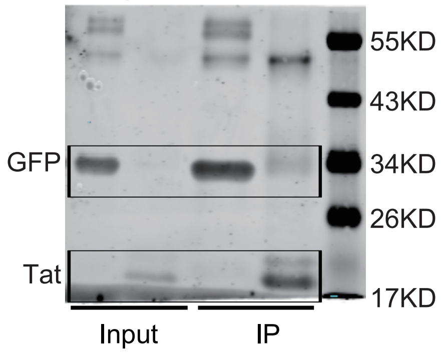

Fig 2d

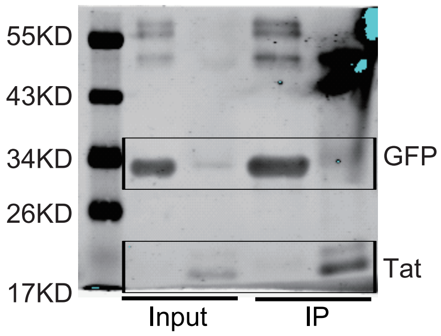

Supplementary Fig 4a

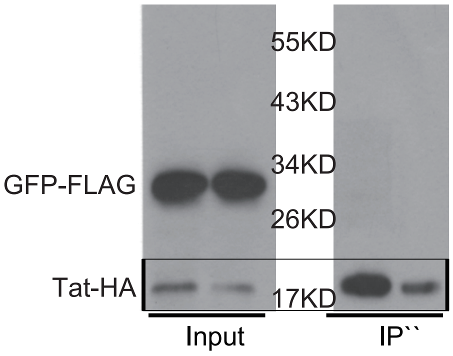

Fig 2e

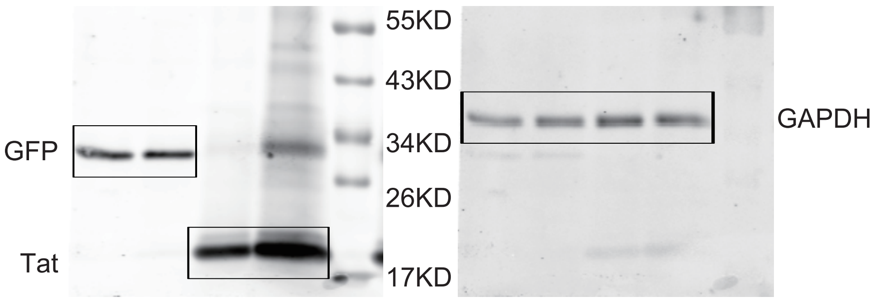

Fig 2f

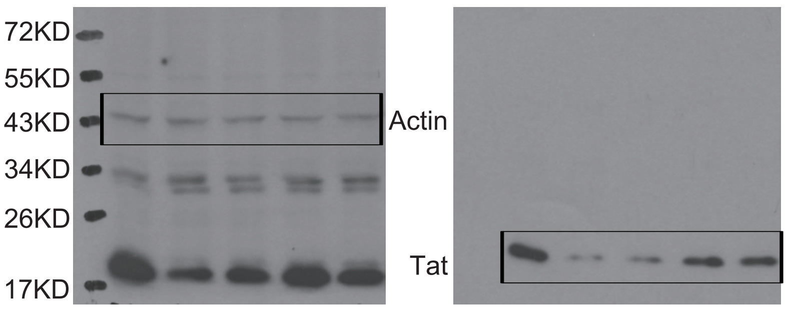

Fig 3b

Supplementary Figure 6

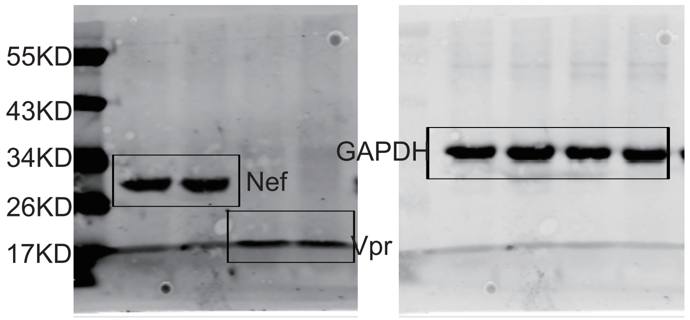

Supplemrntary Fig 4c

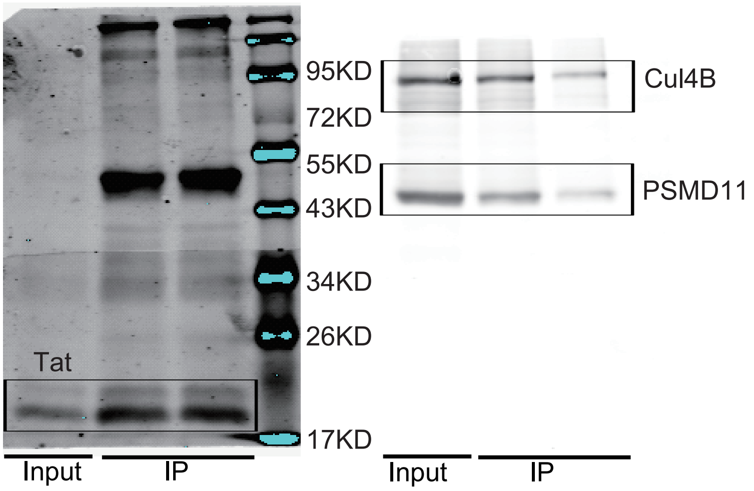

Supplemrntary Fig 4e

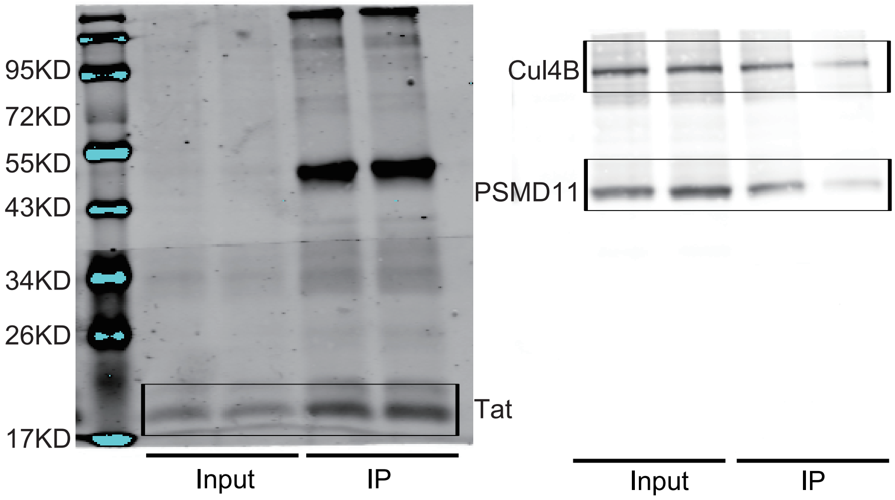

Supplemrntary Fig 4f

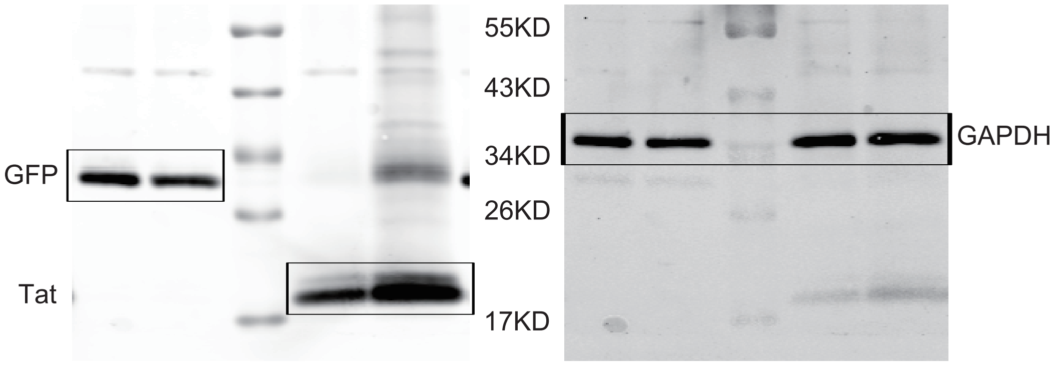

Fig 4b

Supplementary Figure 6 Continued

Supplementary Figure 6 Uncropped blots scans used in the manuscript. Boxes indicate approximate portions of the films that are included in the figures. Below the blots it is indicated to which figure they correspond.

Supplementary Table 1

Table S1 Primer sets for constructs and primers for site direct mutations or deletions on HIV-1 Promoter (Red characters indicate the mutated sites)			
Contructs or mutants	Forword	Reverse	Discription
pHIV-Pro-Luc	CGGGGTACCTGGAAGGGCTAATTTGGTCCCA	CGCGGATCCTGCTAGAGATTTTCCACACTGA	Full lenth of HIV-1 NL4-3 5' LTR sequence
pcDNA3.1-NRON	AGCCCAAGCTTCACATCTCTAATGTAAACAACCCAGC	CGGGGTACCGGAAAAAATTTCTCCTTAACTATTTC	Full lenth of human NRON sequence
pHIV-Pro-mNFAT-1	GACTTTGAGCTGGGGACTTTGAAGGGAGGTGTGGCCTGGGCG	TCAAAGTCCCCAGCTCAAAGTCCCTTGTAGAAAGCT	nt 351 to 380: 5'-GGACTTTGAGCTGGGGACTTTGAAGGGAGG-3'
pHIV-Pro-mNFAT-2		CGCGGATCCTGCTAGAGATTTAGAACACTGACTAAAAGGGTC	nt 607 to 634: 5'-TTTTAGTCAGTGTTCTAAATCTCTAGCA-3'
pHIV-Pro-Core	CGGGGTACCGGGAGGTGTGGCCTGGGCGGGACT	CGCGGATCCGGGTTCCCTAGTTAGCCAGAGAGC	nt 374 to 513 of HIV-1 NL4-3 5' LTR sequence
pHIV-Pro-dTAR	GGGTCTCTCTGGTTAGTCTGGCTAACTAGGGAACCC	TCCCTAGTTAGCCAGACTAACCAGAGAGACCCAGTAC	Deletion of nt 470 to 492 of HIV-1 NL4-3 5' LTR sequence
pHIV-Pro-dTAR/mU	GGTTAGACCAGAACAGAGCCTGGGAGCTCTCTGGC	AGAGCTCCCAGGCTCTGTTCTGGTCTAACCAGAGAGA	nt 466 to 489: 5'-TTAGACCAGAACAGAGCCTGGGAG-3'
pMMLV-Pro-Luc	CGGGGTACCAATGAAAGACCCCACCTGTAGG	CGCGGATCCAATGAAAGACCCCCGCTGACG	Full lenth of MMLV Promoter sequence
pRSV-Pro-Luc	CGGGGTACCCTGCTCCCTGCTTGTGTGTTG	CGCGGATCCGGTGCACACCAATGTGGTGAA	Full lenth of RSV Promoter sequence
Tat exon 1	CGCGGATCCGCCACCATGGAGCCAGTAGATCCTAGACT	GGGATTGGGAGGTGGGTTGCTTTGATAGAGAAACTTGATG	

Tat exon 2	TTCTCTATCAAAGCAACCCACCTCCCAATCCCGAGGGGAC	CCGCTCGAGCTAGGCGTAGTCGGGCACGTCGTAGGGGTAATCGAATGGATCTGTCTCTG	With HA epitope sequence tagged at the C' terminus
pcDNA3.1-CUL4B-FLAG	**CGCGGATCCGCCACCATGATGTCACAGTCATCTGGAT**	CCGCTCGAGTTACTTGTCATCGTCATCCTTGTAATCTCCACCTGCAATATAGTTGTACTGGTTTGGAT	Full lenth of human CUL4B cDNA sequence with FLAG epitope sequence tagged at the C' terminus
pcDNA3.1-PSMD11-FLAG	**CGCGGATCCGCCACCATGGCTGCAGCGGCGGTGGTGGAGTT**	CCGCTCGAGTTACTTGTCATCGTCATCCTTGTAATCTCCACCTGTCAGTTTCTTGGCTTTGTTGTAG	Full lenth of human PSMD11 cDNA sequence with FLAG epitope sequence tagged at the C' terminus
pFLAG-Ub	CGGAATTCGCCACCATGGATTACAAGGATGACGATGACAAGATGCAGATCTTCGTGAAAACCC	**TGCTCTAGATAACCACCTCTCAGACGCAGG**	Full lenth of human ubiquitin cDNA sequence with FLAG epitope sequence tagged at the C' terminus

Supplementary Table 2

Table S2 Target Sequences of siRNAs agianst lncRNAs used in the study			
lncRNA	Target Sequence 1	Target Sequence 2	Target Sequence 3
NRON	CTGTTTCCACTACTGCTCC	ATCATTAGGCTAATAACGC	ATACTGTTTCCACTACTGC
AC004840	GCACCTTGGAGATGTCATT	GGACAGAGTGCAAGGACAT	
SNHG1	GCCAGCACCTTCTCTCTAA	GAGGACATCAGAAGGTGAA	
linc00630	GCTTCTACTCCTGACTTAA	GCGGTATACAACCACACTA	
TUG1	CCTCAAGCCAAATAGCTAA	GCCTCTATTCCTGTATGTA	

Supplementary Table 3

Table S3 Primer sets for real-time RT-PCR used in the study		
Gene or lncRNA name	Forword	Reverse
Beta-actin	GCATGGAGTCCTGTGGCA	CAGGAGGAGCAATGATCTTGA
GAPDH	CGCTCTCTGCTCCTCCTGTT	CCATGGTGTCTGAGCGATGT
NRON	ACGTTCCTTAATGTACGCCTTTGC	TTGGCCGTGTCCTGAGTCCTT
LINC00489	CCTGCTCGATGCACCTCT	CACAGTCCCTGCCGTCTT
AC004840	AGACCCCATACAGCAGCGACCA	CACGCTGACCCGAAATCCTCCA
RP11-86H7	TGCCTCATTATCCCCGGATGCCT	TGACACTCCCAGGACTTGACAAAGC
RP11-62I21	ATGGAACAGAGGTGGCCTACGGTC	GAACCGTCATGGCTGCTTTGAGGA
SNHG6	GAGCCGTTAGTCATGCCGGTGT	GCTCAATACATGCCGCGTGATCCT
SNHG1	TGCCTCCCAGGGTGAATACAGGT	AGAGAGAAGGTGCTGGCCCTTTG
SCAND2	CAGAAAACGTCCAGTGCAGGTGGG	GTGCCACCTGCTTCTCAAGTCCTG
RP11-67K19	CCCCAGAACAATGCTGGAGGCAA	AGCAGGCCATTTGTCCAGTGAGAGA
LINC00900	GGGAAGTGCCAGGATGGGGTATCA	TCAACCTGGTTCACTCCAGGCGAA
LINC00502	AGTAGCAGGCTTGGGGACGTTTTC	TGGTGCTGTCACTTCACACTGCTTG
RP11-418J17	ACACTGCCCCATCTTGAATCGCC	ACACCAGTCACCTTAGCGGCAC
LINC00426	TTCTGCAGCCTCCCTTGTTAGCCA	AACTGTTGCGACATTGTTGCGGG
LINC00630	TAACCCAGTGCCTCGTATGGTGCC	GGAATCAGGCATCACATCCCACCA
AP000402	ATCCAGGCCTGCTTTGGATGTCTT	AAGGGCGTGTACAATCAAGGCAGT
LINC00491	TCAGCCCATCACGAGGTTGCTT	AGACCTTTGGCCTCTTTTGGGGT
NEAT1	CAGAGCCCATGAATGCCAGCAG	ACGCACAAGAAGGCAGGCAAACA
THAP7-AS	GTTAGTAAGCGGCGCGAGCGTG	CATCGTGCCGGGGTGTTTACGG
Gas5	TCTTGCCTCACCCAAGCTAGAG	TTGTGCCATGAGACTCCATCAG
TUG1	TAGCAGTTCCCCAATCCTTG	CACAAATTCCCATCATTCCC
T-bet	TGGTTGGGGAAGTGGGGCTCAA	TGCTCCCATGTCCCACACCTGA
Firefly Luciferase	CAACACCCCAACATCTTCGACGC	GGACTTTCCGCCCTTCTTGGCCTT

HIV-1 total mRNA (Nef-3'LTR)	GGTGGGTTTTCCAGTCACACCTCAG	GCACCATCCAAAGGTCAGTGG
HIV-1 Tat	CGCGGATCCGCCACCATGGAGCCAGTAGATCCTAGACT	GGGATTGGGAGGTGGGTTGCTTTGATAGAGAAACTTGATG
CUL4B	CCCCTCCCCGAGGCTCTTAATTCT	CCACGTACACAGGGAAAAGAGCACA
PSMD11	GGGCTTCCAAGCAAGTCCAGACA	ACTGGACCATGCTGCCCTGGAA
HUWE1	GCCCTCGAAGGCATGAATGGCA	GCCAACAGTAGCATGTGGCGGA
